# Efficient Multi-Scale Stereo-Matching Network Using Adaptive Cost Volume Filtering

**DOI:** 10.3390/s22155500

**Published:** 2022-07-23

**Authors:** Suyeon Jeon, Yong Seok Heo

**Affiliations:** 1 Department of Artificial Intelligence, Ajou University, Suwon 16499, Korea; suyeon1804@ajou.ac.kr; 2 Department of Electrical and Computer Engineering, Ajou University, Suwon 16499, Korea

**Keywords:** deep learning, stereo matching, knowledge distillation, cost volume filtering, lightweight network

## Abstract

While recent deep learning-based stereo-matching networks have shown outstanding advances, there are still some unsolved challenges. First, most state-of-the-art stereo models employ 3D convolutions for 4D cost volume aggregation, which limit the deployment of networks for resource-limited mobile environments owing to heavy consumption of computation and memory. Although there are some efficient networks, most of them still require a heavy computational cost to incorporate them to mobile computing devices in real-time. Second, most stereo networks indirectly supervise cost volumes through disparity regression loss by using the softargmax function. This causes problems in ambiguous regions, such as the boundaries of objects, because there are many possibilities for unreasonable cost distributions which result in overfitting problem. A few works deal with this problem by generating artificial cost distribution using only the ground truth disparity value that is insufficient to fully regularize the cost volume. To address these problems, we first propose an efficient multi-scale sequential feature fusion network (MSFFNet). Specifically, we connect multi-scale SFF modules in parallel with a cross-scale fusion function to generate a set of cost volumes with different scales. These cost volumes are then effectively combined using the proposed interlaced concatenation method. Second, we propose an adaptive cost-volume-filtering (ACVF) loss function that directly supervises our estimated cost volume. The proposed ACVF loss directly adds constraints to the cost volume using the probability distribution generated from the ground truth disparity map and that estimated from the teacher network which achieves higher accuracy. Results of several experiments using representative datasets for stereo matching show that our proposed method is more efficient than previous methods. Our network architecture consumes fewer parameters and generates reasonable disparity maps with faster speed compared with the existing state-of-the art stereo models. Concretely, our network achieves 1.01 EPE with runtime of 42 ms, 2.92 M parameters, and 97.96 G FLOPs on the Scene Flow test set. Compared with PSMNet, our method is 89% faster and 7% more accurate with 45% fewer parameters.

## 1. Introduction

Estimating depth from a stereo image has been a fundamental and classic computer vision problem for decades [1]. Stereo matching aims to estimate the correspondence between the pixels of a rectified stereo image pair. If a pixel at (x,y) in the reference left image matches a pixel at (x−d,y) in the target right image, the horizontal difference *d* between the corresponding pixels is a disparity. Using disparity *d*, camera focal length *f*, and distance between cameras *B*, the depth of the pixel can be calculated as $\frac{fB}{d}$. Because 3D depth information is essential for various real-world applications, including robot navigation [2], augmented/virtual reality (AR/VR) [3,4], autonomous driving for vehicles [5], and network security domains [6,7], reliable real-time stereo matching processing in restricted hardware environments is important.

Since the introduction of the seminal work known as MC-CNN [8], convolutional neural networks (CNNs) have been used to learn strong feature representation [8,9] to compute the cost of corresponding patches in the input stereo image pair. Consequently, they achieve more significant increases in accuracy than traditional stereo matching algorithms. However, because most of them adopt handcrafted cost aggregation methods and do not realize the fully end-to-end networks, they suffer from errors for ambiguous regions such as textureless regions.

DispNet [10] presented the first end-to-end stereo-matching network by building a 3D cost volume with a correlation layer followed by 2D convolution layers to estimate a disparity map and showed significant improvement in accuracy compared with previous patch-wise CNNs [8,9]. Instead of the 3D cost volume with the correlation layer, refs. [11,12] built a 4D cost volume by concatenating the left and right feature maps along the disparity levels. Subsequently, by processing the cost volume through a series of 3D convolution layers, they achieved better accuracy than 3D cost-volume-based methods. Although these networks that process 4D cost volume with 3D convolutions achieved state-of-the-art accuracy, heavy computation and memory consumption prevent them from running in mobile environments and real-time processing.

To address this problem, various efficient stereo-matching networks [13,14,15,16,17] have recently been proposed. Although these networks have significantly improved performance in terms of efficiency compared with previous networks, they still require a heavy computational cost to incorporate them to mobile computing devices in real time. Even though some of them are sufficiently light to run in mobile environments, there is a significant decrease in accuracy.

On the other hand, since DispNet [11] proposed the softargmin function that robustly calculates continuous disparity values with subpixel precision from a cost distribution, it has been adopted in most stereo-matching networks to regress a continuous disparity map. Most of them trained their networks in an end-to-end manner by defining the loss function using the difference between the predictive disparity map and the ground truth disparity map. The networks learn the cost distribution in an indirect supervision manner by reducing this loss function. Because this indirect supervision results in many possibilities for unreasonable cost distributions as long as the result after regression is correct, learning with these flexible cost volumes leads to overfitting [18]. Thus, direct supervision is required for cost volume regularization with probability distribution which peaks at the true disparity especially at the pixels where a multi-modal probability distribution is predicted, such as the boundaries of the object [19].

There are a few works that handle this problem. AcfNet [18] adds a direct constraint to the predicted cost volume by generating a unimodal probability distribution that peaks at the ground truth disparity and filters the predicted cost volume through it. CDN [19] addresses the problem by directly taking the mode as a predicted disparity value without a regression function, such as the softargmin operation. However, these methods still perform insufficient regularization for the cost volume because the artificial cost distribution generated using only the ground truth disparity value does not contain information such as similarity among different disparities [20,21,22,23].

In this paper, to solve the problems mentioned above, we propose a new efficient network architecture called a Multi-scale Sequential Feature Fusion Network (MSFFNet) and a new loss function called adaptive cost-volume-filtering (ACVF) loss for direct cost volume supervision. MSFFNet consists of a proposed Multi-scale Sequential Feature Fusion (MSFF) module which connects SFF modules [17] with different scales in parallel for an efficient and accurate multi-scale network architecture. For higher efficiency, only the smallest scale in the MSFF module is processed for the entire disparity search range. The other scales are processed for the odd disparities among the entire disparity range. In addition, the cross-scale fusion [24] is adopted at the end of the MSFF module to compensate for the lack of scale information and allow each cost volume with different scale to interact with each other. In addition, to effectively combine the multi-scale cost volumes generated from different scales, we present an interlaced cost concatenation method.

**Figure 1:**
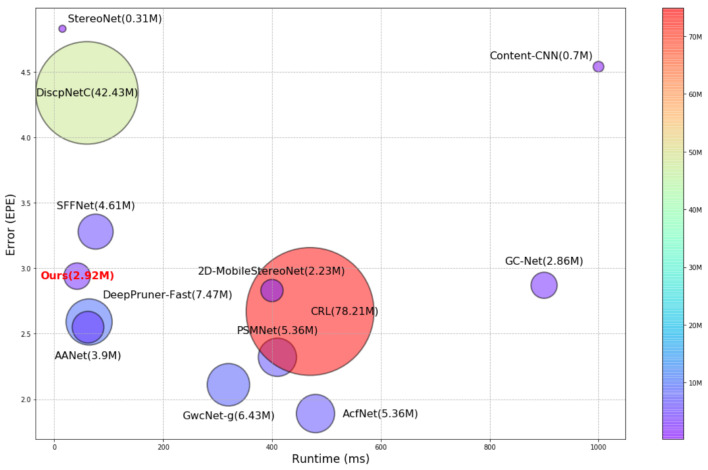
Visualization of runtime, accuracy, and number of parameters of stereo-matching networks, including Content-CNN [9], GC-Net [11], PSMNet [12], StereoNet [13], GwcNet-g [15], DeepPruner-Fast [26], AANet [16], AcfNet [18], DispNetC [10], CRL [27], SFFNet [17], 2D-MobileStereoNet [28], and our proposed method, on KITTI 2015 leader board [25].

Moreover, the proposed ACVF loss adds a constraint to the estimated cost distribution, using both the ground truth disparity map and the probability distribution map generated from the teacher network with higher accuracy. Thus, a unimodal distribution is generated with the ground truth disparity value [18], and the distribution generated from the teacher network is utilized for knowledge distribution [21,22,23] which transfers the *dark knowledge* of the cumbersome teacher network to our network as student. However, there are some pixels for which the teacher network is not accurate and that negatively affect the student network. To avoid the negative effects of the distillation [22], the distribution from the teacher network is adaptively transferred using the ratio between the errors of the teacher and our proposed networks for each pixel. Because the proposed loss function does not require additional parameters and computations for inference, it allows us to consider the effectiveness of the direct supervision of the cost volume while maintaining the efficiency of our network. As shown in Figure 1, our proposed network predicts a reasonably accurate disparity map with an extremely fast runtime using small numbers of parameters compared with other efficient stereo-matching networks.

The main contributions of this paper are summarized as follows:

1. We propose an efficient multi-scale stereo-matching network, MSFFNet, that connects multi-scale SFF modules in parallel and effectively generates a cost volume using the proposed interlaced cost concatenation method.
2. We propose an adaptive cost-volume-filtering loss that adaptively filters the estimated cost volume using a ground truth disparity map and an accurate teacher network for direct supervision of the estimated cost volume.
3. We achieved competitive results on the Scene Flow [10] and KITTI-2015 [25] test sets. The proposed MSFFNet has only 2.92 M parameters with a runtime of 42 ms, which shows that our method is more efficient and reasonably accurate compared with other stereo-matching networks.

The following sections of this paper are organized as follows. We introduce several related works in Section 2. Section 3 introduces the details about methodology and implementation of our proposed method. Various experiments including comparative results with previous methods and ablation studies are demonstrated in Section 4. Finally we conclude the paper in Section 5.

## 2. Related Works

Our proposed method includes an efficient network architecture for stereo matching and a new loss function which filters the cost volume for increasing accuracy. Thus, in this section, the relevant works about efficient stereo-matching networks and cost volume filtering are separately discussed. Table 1 summarizes the representative related works in terms of types of cost volume, network architecture, cost volume filtering, and datasets used for training such as Scene Flow [10] and KITTI [25,29].

### 2.1. Efficient Stereo-Matching Networks

Since the first end-to-end training for a stereo-matching network [10] was proposed, stereo-matching networks usually generate a single-scale cost volume for matching cost computation. Refs. [10,27,33,34] constructed a 3D cost volume using a correlation operation between corresponding left and right features. Although a large amount of useful feature information for cost aggregation is lost through correlation, there is an advantage in terms of computational cost because the generated 3D cost volume is processed using 2D convolutions. Meanwhile, refs. [8,12] generated a 4D cost volume by concatenating two corresponding features and achieved higher accuracy compared with 3D cost-volume-based methods. However, 3D convolutions are required for processing the 4D cost volume, leading to a significant increase in computing resources and runtime.

There is a trade-off between efficiency and accuracy according to the choice of the dimension of the cost volume. Thus, various efficient stereo-matching networks have been studied extensively in recent years [14,16]. To efficiently process the 4D cost volume with 3D convolutions, several networks adopted a coarse to fine architecture that leverages multi-scale pyramid cost volumes. This method can enhance accuracy without expensive computational complexity and memory consumption. Moreover, multi-scale cost volumes are more sufficient to utilize spatial information of the stereo image pair. These networks using multi-scale cost volumes are classified as sequential method and parallel method according to the type of connecting multi-scale feature maps.

Sequential method networks [14,31,32] generate and aggregate the cost volume over the full disparity range only at the initial coarsest scale. Then, to correct the error of the disparity map generated from the previous scale, the succeeding networks warp the right feature map using the disparity map and process the residuals that cover only the offset range of each disparity search range. The rough disparity map from the network that covers the coarse scale is iteratively refined through the succeeding networks towards the bottom of the pyramid in the original resolution by adding the residual to the previous disparity result. Because the computational complexity of cost aggregation with 3D convolution increases cubically with resolution and linearly with disparity range, this sequential method considerably reduces the computational cost and memory consumption. However, the sequential method has the following disadvantages. The cost aggregation conducted only at the first stage with the smallest resolution is insufficient to estimate accurate disparity for sharp regions. In addition, the refinement using the networks of succeeding stages with respect to the offset range can propagate errors when the error is larger than the offset.

Parallel method networks [16,30] perform aggregation and estimate the disparity map at each scale of the multi-scale cost volume. These methods can capture both robust global features and detailed local feature representations by integrating all the different scales of cost volumes. Parallel method networks regularize cost volumes and predict disparity maps with high accuracy by utilizing these rich representations. Although there is an advantage in terms of accuracy, they still require a large amount of computation because the cost aggregation is performed at all scales, even though it has decreased compared with the single scale-based networks [10,17].

Our network efficiently combines the sequential and parallel methods. To make our network more efficient than other parallel networks, we process the entire disparity search range only at the initial coarsest scale similar to the sequential methods. The other higher scales are processed only half of the full disparity range. In addition, using a new interlaced concatenation method, we effectively combine the multi-scale cost volumes which include the entire disparity range.

### 2.2. Cost Volume Filtering

Most deep-learning-based stereo-matching networks use loss functions based on the difference between the true and predicted disparities because they consider stereo matching as a regression problem. However, there are only a few methods that supervise the cost volume. Because a disparity is the regression result from the cost volume, learning cost volume indirectly through the disparity regression loss easily causes an overfitting problem [18,19], especially around the edge regions.

To mitigate this problem, the cost volume filtering for direct constraints on the cost volume was first proposed in AcfNet [18]. Through the addition of a cost-volume-filtering module, which filters the estimated cost volume with a unimodal distribution that peaks at the ground truth disparity, direct supervision of the cost volume is conducted. CDN [19] also addresses this problem by proposing a new network architecture for stereo matching that can estimates arbitrary disparity values. Thus, the mode value can be directly chosen as a disparity without a regression step.

Unlike refs. [18,19] which uses the artificially generated cost distributions using the ground truth disparity map, we perform direct supervision to the cost volume through a loss function which includes two kinds of filtering using the cost volume from the ground truth disparity map and that estimated from the teacher network with higher accuracy via knowledge distillation.

## 3. Proposed Method

**Figure 2:**
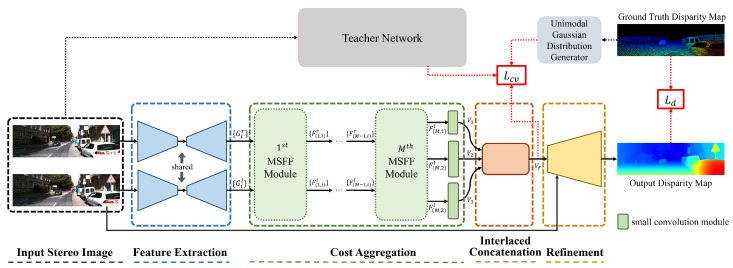
An overview of the proposed method. A path using dotted arrows is required only for the training phase.

Figure 2 presents an overview of the proposed network. Given a rectified stereo image pair, each image is inserted into a U-Net [35] feature extractor to generate multi-scale feature maps. Using these feature maps as inputs, a cost aggregation module aggregates the matching costs and generates multi-scale cost volumes, where a MSFF module is proposed to efficiently generate them by connecting the SFF [17] modules of different scales in parallel. The final cost volume for disparity regression is obtained by concatenating the cost volume using the proposed interlaced concatenation method. Finally, an initial disparity map is regressed with the softargmax function from the cost volume and hierarchically upsampled and refined with a refinement network [13] to produce a final disparity map. To train the network, we define a loss function that consists of a disparity regression loss and an adaptive cost-volume-filtering (ACVF) loss. Specifically, to supervise the cost volume, the proposed ACVF utilizes both the ground truth disparity map and probability distribution map generated from a teacher network with higher accuracy. Detailed explanations of each part are provided in the following subsections.

### 3.1. Feature Extractor

Given a rectified stereo image pair $I_{l}\in R^{W\times H\times 3}$ and $I_{r}\in R^{W\times H\times 3}$, each image is inserted into a U-Net [35] feature extractor, to generate feature maps ${\left\{G_{i}^{l}\in R^{W_{i}\times H_{i}\times C_{i}}\right\}}_{i=1}^{3}$ and ${\left\{G_{i}^{r}\in R^{W_{i}\times H_{i}\times C_{i}}\right\}}_{i=1}^{3}$, respectively. Here, *i* represents the scale index, and $W_{i}$ and $H_{i}$ are $1/2^{\left(5-i\right)}$ times the width and height of the original input image, respectively. The channel size $C_{i}$ is fixed to 32 for all scales. Similar to [14,16], $G_{i}^{l}$ and $G_{i}^{r}$ are extracted from the different positions of the decoder of the feature extractor network, where the corresponding spatial resolutions of the feature maps for each scale are 1/16, 1/8, and 1/4 times the original spatial resolution of the input image, respectively.

### 3.2. Cost Aggregation

**Figure 3:**
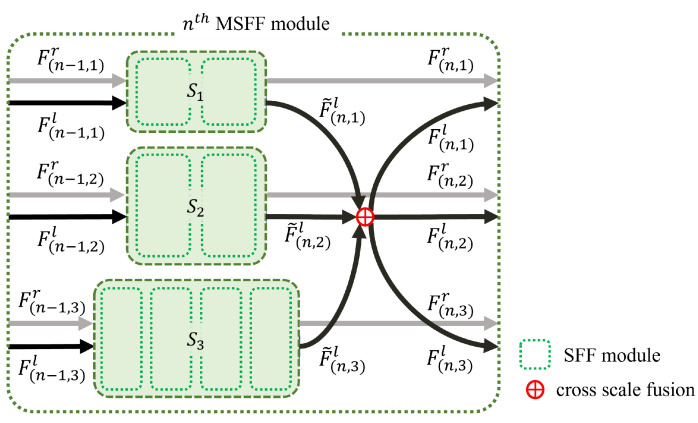
Details of the proposed MSFF module.

**Figure 4:**
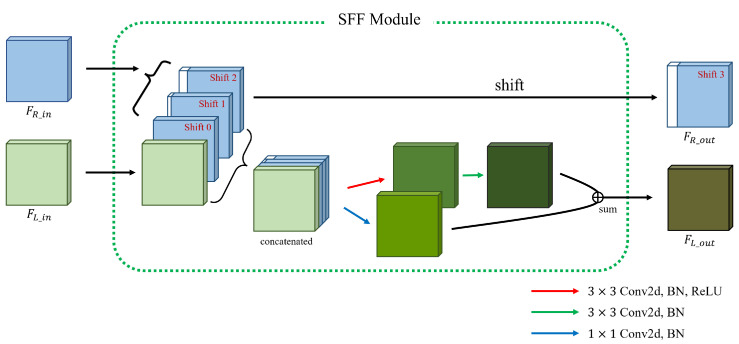
Details of a single SFF module in the $S_{1}$.

**Figure 5:**
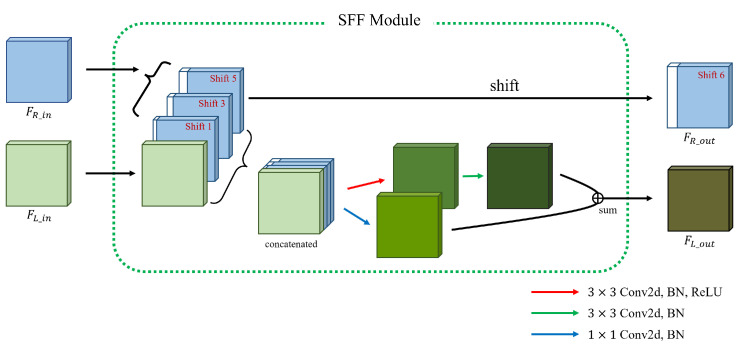
Details of a single SFF module in the $S_{2}$ and $S_{3}$.

From the multi-scale feature maps ${\left\{G_{i}^{l}\right\}}_{i=1}^{3}$ and ${\left\{G_{i}^{r}\right\}}_{i=1}^{3}$ obtained using the feature extractor module, the proposed cost aggregation module generates various cost volumes ${\left\{V_{i}^{}\right\}}_{i=1}^{3}$ with different spatial resolutions. To this end, we propose an MSFF module that acts as a building block for the cost aggregation module. Concretely, the cost aggregation module consists of a series of *M* MSFF modules followed by a small convolution layer. As shown in Figure 3, each MSFF module consists of combined SFF modules [17] ${\left\{S_{i}\right\}}_{i=1}^{3}$ with different scales and a cross-scale fusion operation that combines them so that they share different scale information. Specifically, $n^{th}$ MSFF module generates feature maps ${\left\{F_{\left(n,i\right)}^{l}\in R^{W_{i}\times H_{i}\times 32}\right\}}_{i=1}^{3}$ and ${\left\{F_{\left(n,i\right)}^{r}\in R^{W_{i}\times H_{i}\times 32}\right\}}_{i=1}^{3}$ which are inserted to the next $n+1^{th}$ MSFF module from ${\left\{F_{\left(n-1,i\right)}^{l}\right\}}_{i=1}^{3}$ and ${\left\{F_{\left(n-1,i\right)}^{r}\right\}}_{i=1}^{3}$, where the input of the first MSFF module is the feature maps ${\left\{G_{i}^{l}\right\}}_{i=1}^{3}$ and ${\left\{G_{i}^{r}\right\}}_{i=1}^{3}$, that is, $F_{\left(0,i\right)}^{l}=G_{i}^{l}$ and $F_{\left(0,i\right)}^{r}=G_{i}^{r}$. In the $n^{th}$ MSFF module, $S_{i}$ generates the intermediate feature maps ${\tilde{F}}_{\left(n,i\right)}^{l}$ and ${\tilde{F}}_{\left(n,i\right)}^{r}$ from $F_{\left(n,i\right)}^{l}$ and $F_{\left(n,i\right)}^{r}$. To prevent heavy computations and memory consumption, only $S_{1}$ with the smallest scale processes the entire disparity range, whereas the others, including $S_{2}$ and $S_{3}$, process only odd disparities of the full search range. In more detail, only $S_{1}$ processes the entire disparity range, which amounts to 1/16 of the maximum disparity range through two SFF modules [17]. Thus, as shown in Figure 4, the input right feature $F_{\left(n,1\right)}^{r}$ is shifted by only one pixel to the right at a time in the SFF module. However, as shown in Figure 5, $S_{2}$ and $S_{3}$ process only odd disparities by shifting $F_{\left(n,2\right)}^{r}$ and $F_{\left(n,3\right)}^{r}$ 2 pixels to the right, respectively, unlike the original SFF module [17]. This reduces the computation and parameters of $S_{2}$ and $S_{3}$ by half compared with processing the entire disparity range at that scale.

Next, the output feature map $F_{\left(n,s\right)}^{l}$ of scale *s* in the *n*th MSFF module is obtained by fusing the intermediate feature maps ${\left\{{\tilde{F}}_{\left(n,i\right)}^{l}\right\}}_{i=1}^{3}$ with the cross-scale fusion function f(·), which is defined as

(1) $$F_{\left(n,s\right)}^{l}=\sum_{i=1}^{3}f_{i}^{s}\left({\tilde{F}}_{\left(n,i\right)}^{l}\right),$$

where $f_{i}^{s}\left(\cdot \right)$ is a function for the adaptive fusion of multi-scale features, similar to [24], that is defined as

(2) $$f_{i}^{s}=\left\{\begin{array}{cc} \begin{array}{cc} I, & i=s, \end{array}\\ (s-i)\cdot strided\;3\times 3\;convs, & i>s,\\ upsampling\;1\times 1\;conv, & i<s, \end{array}\right.$$

where I is an identity function, (s−i)·strided3×3convs represents a series of (s−i) numbers of 3×3 convolutions with stride 2 for $2^{\left(s-i\right)}$ times downsampling to make the scale consistent, and upsampling1×1conv represents bilinear upsampling for scale consistency, then 1×1 convolution is followed. It is noteworthy that $f_{i}^{s}\left(\cdot \right)$ fuses the features from different scales to share their information. By fusing these feature maps, some information about absent disparity is compensated using those of other scales. That is, this fusion is conducted so that each feature map obtains global and robust semantic information which covers broader spatial and disparity ranges from features of smaller scale. Furthermore, precise and local information for the fine detail is compensated from features of larger scale.

**Figure 6:**
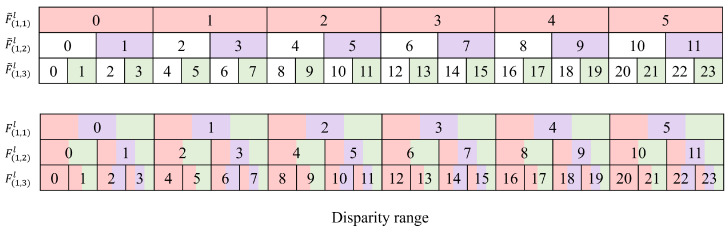
Details of multi-scale features before and after cross-scale fusion in the disparity domain.

Figure 6 illustrates the disparity range covered in the first MSFF module, where bins with white color represent absent disparity values that are not included in the feature maps. A disparity value *d* in ${\tilde{F}}_{\left(n,1\right)}^{l}$ corresponds to {2d,2d+1} for ${\tilde{F}}_{\left(n,2\right)}^{l}$ and corresponds to {4d,4d+1,4d+2,4d+3} for ${\tilde{F}}_{\left(n,3\right)}^{l}$ owing to scale difference. For the first MSFF module, the feature map from $S_{1}$ module includes the disparity range of [0,5] on the 1/16 scale because each SFF module in $S_{1}$ covers three disparity values, and two SFF modules are serially connected. Similarly, $S_{2}$ covers [0,11] disparity range in the 1/8 scale using two SFF modules without even disparity values. Similarly, $S_{3}$ covers the disparity range of [0,23] on the 1/4 scale with only odd disparity values. As shown in Figure 6, after the cross-scale fusion process, there is not absent disparity value for each feature map $F_{\left(n,i\right)}^{l}$, which compensates for the lack of even values of disparity information by fusing information from different scales.

Note that the left output feature map $F_{\left(M,i\right)}^{l}$ of the last MSFF module contains the aggregated cost distribution through the property of the SFF module [17]. Thus, the output cost volume $V_{i}$ is obtained using a series of two 3×3 convolutions from $F_{\left(M,i\right)}^{l}$ to render the channel dimension of $V_{i}$ equal to the disparity range at that scale. Specifically, the channel number of the output cost volume $V_{1}$ is the same as the full disparity range for its own scale, whereas those of $V_{2}$ and $V_{3}$ are half of the disparity range at those scales.

### 3.3. Interlaced Cost Volume

**Figure 7:**
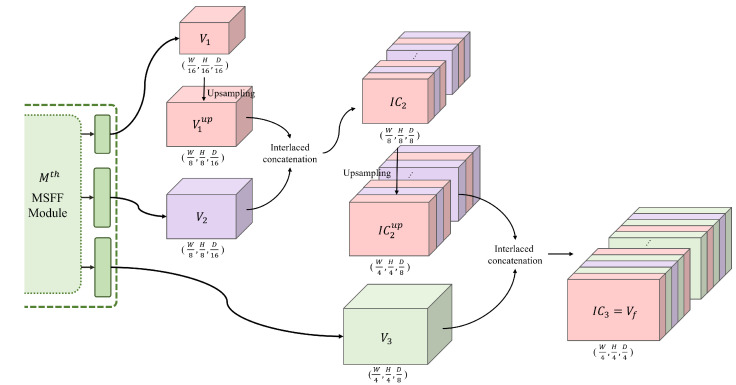
Process of interlaced concatenation.

We propose an interlaced concatenation method to combine the multi-scale cost volumes ${\left\{V_{i}\in R^{W_{i}\times H_{i}\times D_{i}}\right\}}_{i=1}^{3}$ and produce the final cost volume $V_{f}$ for disparity regression. Here, *i* represents the scale index, and $W_{i}$ and $H_{i}$ are $1/2^{\left(5-i\right)}$ times the width and height of the input image $I_{l}$, respectively. $D_{1}$, $D_{2}$, and $D_{3}$ are 1/16, 0.5×(1/8), and 0.5×(1/4) times the maximum disparity search range, respectively. As shown in Figure 7, ${\left\{V_{i}^{up}\in R^{W_{i+1}\times H_{i+1}\times D_{i}}\right\}}_{i=1}^{3}$ is obtained by upsampling the cost volume $V_{i}$ only in the spatial domain to achieve the same spatial resolution as $V_{i+1}$ while maintaining the channel dimension. Then, the upsampled $V_{i}^{up}$ and $V_{i+1}$ are interlaced along the channel direction to generate an interlaced cost volume $IC_{i+1}$, where the value of $IC_{i+1}\left(x,y,c\right)$ of (x,y) spatial position and $c^{th}$ channel is defined by

(3) $$IC_{i+1}\left(x,y,c\right)=\left\{\begin{array}{c} \begin{array}{cc} V_{i}^{up}\left(x,y,n\right), & c=2n \end{array}\\ \begin{array}{cc} V_{i+1}\left(x,y,n\right), & c=2n+1 \end{array} \end{array}\right.,$$

where *n* denotes the channel index of $V_{i}^{up}$ and $V_{i+1}$. When the interlaced concatenation is performed, the disparity channels from the lower scale are placed at the even number disparity. The final cost volume $V_{f}$, which is also $IC_{3}$, can be generated in the same manner using $IC_{2}$ and $V_{3}$.

### 3.4. Disparity Regression and Refinement

For each pixel, $V_{f}$ contains a $D_{max}$-length vector that contains the matching costs of the disparity range $D_{max}$. This vector is converted to a probability vector using the softmax operation, where the probability ${\hat{p}}_{d}$ of disparity *d* is defined as

(4) $${\hat{p}}_{d}=\frac{\exp(c_{d})}{\sum_{i=0}^{D_{\max}-1}\exp(c_{i})},$$

where $c_{d}$ is the matching cost in $V_{f}$ for disparity *d*. To estimate an initial disparity map $\hat{D}$ that contains a continuous disparity value for each pixel, the following softargmax function [11] is applied:

(5) $$\hat{d}=\sum_{d=0}^{D_{max}-1}d\times {\hat{p}}_{d},$$

where ${\hat{p}}_{d}$ is the probability corresponding to a candidate disparity *d*. The estimated disparity $\hat{d}$ is obtained using the weighted summation, as shown in Equation (5). This regression-based formulation can produce more robust disparity values with sub-pixel precision than classification-based stereo-matching methods [11].

To obtain a final disparity map *D* with increased accuracy, we use a refinement module for the initial disparity map $\hat{D}$ similar to [13]. In this module, the initial disparity map is upsampled to the same scale as $I^{l}$ and concatenated with $I^{l}$ as an input to the refinement module, where a series of convolution layers are followed to generate a final disparity map.

### 3.5. Loss Function

As shown in Figure 2, to train the network, we employ two loss functions: a disparity regression loss and an ACVF loss. The loss functions are described in detail in the following subsections.

#### 3.5.1. Disparity Regression Loss

The first loss function is a disparity regression loss $L_{d}$, which is adopted in most networks [12,16,26] and is defined as

(6) $$L_{d}=\frac{1}{N}\sum_{j=1}^{N}U_{s}\left(D^{gt}\left(j\right)-D\left(j\right)\right),$$

where $D^{gt}\left(j\right)$ and D(j) are the ground truth disparity and the estimated disparity for *j*th pixel, respectively. *N* is the total number of pixels in $D^{gt}$ and *D*. The smooth $L_{1}$ loss function $U_{s}$ is widely used owing to its robustness and low sensitivity and is defined as

(7) $$U_{s}\left(x\right)=\left\{\begin{array}{c} \begin{array}{cc} 0.5x^{2}, & if\;\left|x\right|⩽1, \end{array}\\ \begin{array}{cc} \left|x\right|-0.5, & otherwise. \end{array} \end{array}\right.$$

Note that $L_{d}$ in Equation (6) is fully differentiable and useful for smooth disparity estimation.

#### 3.5.2. Adaptive Cost-Volume-Filtering Loss

**Figure 8:**
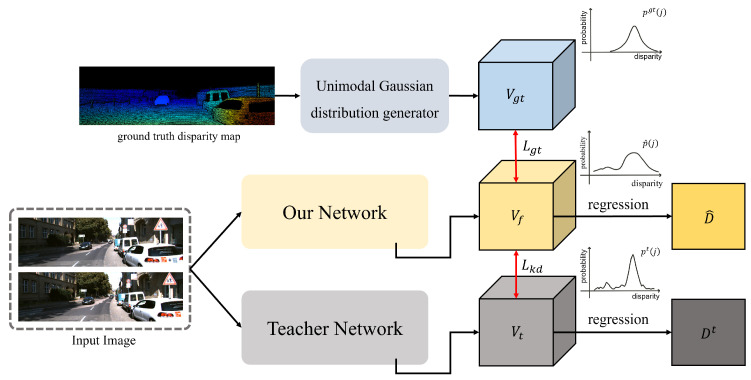
Process of adaptive cost volume filtering.

As shown in Figure 8, to directly supervise the estimated cost volume, we propose to filter the cost volume $V_{f}$ using two types of cost volumes $V_{gt}$, generated using the ground truth disparity map, and $V_{t}$, generated from the teacher network with higher accuracy. The reason for using both types is that the ways cost volumes are generated are different, and the goal of usage is also different. $V_{gt}$ is a unimodal distribution with a peak at the ground truth disparity value; thus, it can help filter the estimated cost volume for reducing the bias error aspect. In contrast, because $V_{kd}$ is the cost volume estimated from the teacher network, it can be helpful in reducing variance errors [22].

##### Cost Volume Filtering Using Ground Truth Disparity

To filter the cost volume $V_{f}$ using the ground truth disparity map, we generate a unimodal cost volume $V_{gt}$ similar to [18]. Given a ground truth disparity map $D^{gt}$, we first downsample it four times to generate ${\hat{D}}^{gt}$ which has the same spatial resolution as $V_{f}$. Then, the unimodal distribution value $p_{d}^{gt}\left(j\right)$ for $j^{th}$ pixel and disparity *d* is defined using the softmax operation as follows:

(8) $$p_{d}^{gt}\left(j\right)=softmax\left(-\frac{|d-{\hat{D}}^{gt}\left(j\right)|}{\sigma }\right),$$

where ${\hat{D}}^{gt}\left(j\right)$ is the ground truth disparity of *j*th pixel, and σ is the variance related to the sharpness of the distribution. From Equation (8), the Laplacian unimodal distribution has a peak near the true disparity value.

With the estimated probability distribution map $\hat{p}$ which is generated from the cost volume $V_{f}$ on the softmax operation and the probability distribution $p^{gt}$ generated from the ground truth disparity map, we define a unimodal cost-volume-filtering loss $L_{gt}$ using the KL-divergence between them, as follows:

(9) $$\begin{array}{cc} L_{gt} & =\frac{1}{N}\sum_{j=1}^{N}KL(p^{gt}\left(j\right),\hat{p}\left(j\right))\\ \; & =\frac{1}{N}\sum_{j=1}^{N}\left(-\sum_{d=0}^{D_{max}-1}p_{d}^{gt}\left(j\right)\cdot \log\frac{{\hat{p}}_{d}\left(j\right)}{p_{d}^{gt}\left(j\right)}\right), \end{array}$$

where $p_{d}^{gt}\left(j\right)$ and ${\hat{p}}_{d}\left(j\right)$ are the probability value of $p^{gt}$ and $\hat{p}$, respectively, for *j*th pixel and disparity *d*. *N* is the total number of pixels in $p^{gt}$ and $\hat{p}$.

##### Cost Volume Filtering Using Knowledge Distillation

Using only the Laplacian unimodal, cost distribution from the ground truth disparity is not sufficient to effectively regularize the cost volume of the proposed network. For some pixels at the boundaries of objects, a multi-modal distribution would be more appropriate to help the network estimate accurate disparity [19]. However, the artificially generated distribution would not contain useful information for non-true disparities such as correlation among different disparities. Unlike [18,19] which uses artificially generated distributions, we perform knowledge distillation [21] to regularize the cost volume. The distribution generated from the teacher network with higher accuracy includes *dark knowledge* even for the *non-true disparities* which is helpful to further regularize the cost distributions for some pixels [20,21,22,23]. Thus, given a probability distribution map from the teacher network, the cost-volume-filtering loss $L_{kd}$ using knowledge distillation via weighted KL-divergence is defined as follows:

(10) $$\begin{array}{cc} L_{kd} & =\frac{1}{N}\sum_{j=1}^{N}\left\{\alpha \left(j\right)\cdot KL(p^{t}\left(j\right),\hat{p}\left(j\right))\right\}\\ \; & =\frac{1}{N}\sum_{j=1}^{N}\left\{\alpha \left(j\right)\cdot \left(-\sum_{d=0}^{D_{max}-1}p_{d}^{t}\left(j\right)\cdot \log\frac{{\hat{p}}_{d}\left(j\right)}{p_{d}^{t}\left(j\right)}\right)\right\}, \end{array}$$

where $p_{d}^{t}\left(j\right)$ and ${\hat{p}}_{d}\left(j\right)$ are the probability values of the teacher network and the estimated cost volume at pixel *j* for disparity *d*, respectively. Here, α(j) is a weighting factor of pixel *j* that is defined similar to [22] as follows:

(11) $$\begin{array}{c} \alpha \left(j\right)=(1-\exp\left(-\frac{L^{s}\left(j\right)}{L^{t}\left(j\right)}\right)),\\ L^{s}\left(j\right)=\left|{\hat{D}}^{gt}\left(j\right)-\hat{D}\left(j\right)\right|,\\ L^{t}\left(j\right)=\left|{\hat{D}}^{gt}\left(j\right)-{\hat{D}}^{t}\left(j\right)\right|, \end{array}$$

where ${\hat{D}}^{t}\left(j\right)$ and $\hat{D}\left(j\right)$ are disparity values for *j*th pixel of ${\hat{D}}^{t}$ and $\hat{D}$ obtained from $V_{t}$ and $V_{f}$ after performing the softargmax operation, respectively. The absolute differences $L^{t}\left(j\right)$ and $L^{s}\left(j\right)$ are calculated at pixel *j* with the ground truth disparity map ${\hat{D}}^{gt}$. The weight α(j) is determined according the ratio of the error of both networks. It is worthy to note that the role of this weighting factor is to avoid the negative effects of the distillation for pixels that the teacher network is less accurate than those of our network as student. At *j*th pixel, when the teacher network predicts a disparity with higher error than that of the proposed network, the weight α(j) decreases, and vice versa. Thus, the knowledge from the teacher network is adaptively transferred using the ratio between the errors of the teacher and our proposed networks for each pixel [22].

Now, the ACVF loss $L_{cv}$ is defined by summing $L_{gt}$ and $L_{kd}$ as follows:

(12) $$L_{cv}=\lambda_{1}\cdot L_{gt}+\lambda_{2}\cdot L_{kd},$$

where $\lambda_{1}$ and $\lambda_{2}$ are the weighting factors for $L_{gt}$ and $L_{kd}$, respectively. Note that $L_{cv}$ is a combination of two cost-volume-filtering losses, $L_{gt}$ and $L_{kd}$, where $L_{gt}$ is the loss between $V_{gt}$ and $V_{f}$, and $L_{kd}$ is the loss between $V_{t}$ and $V_{f}$. This $L_{cv}$ loss is reminiscent of a loss function that uses the soft label with knowledge distillation scheme for the classification problem. Most classification methods usually combine a cross-entropy loss using a one-hot distribution with a ground truth label and a knowledge distillation loss using a teacher network to reduce both bias and variance errors [22]. Unlike in classification problems, to regress continuous disparity values, we use a unimodal distribution generated using ground truth disparity instead of a one-hot distribution. In addition, for each pixel, we adaptively combine the knowledge distillation loss based on the ratio between the accuracies of the teacher and student networks similar to [22].

#### 3.5.3. Total Loss Function

To train the proposed network in an end-to-end manner, we employ two types of losses; the disparity regression loss described in Equation (6) and the ACVF loss in Equation (12). The final total loss $L_{total}$ is a combination of these two losses, defined as

(13) $$L_{total}=L_{d}+\beta \cdot L_{cv},$$

where β is a hyperparameter used to balance the two different losses.

## 4. Experiments and Results

### 4.1. Datasets and Evaluation Metrics

To evaluate the performance of the proposed method, we conducted various comparative experiments using two stereo datasets, Scene Flow and KITTI 2015. In addition, ablation studies were conducted on the Scene Flow dataset with various settings.

1. **Scene Flow** [10]: A large-scale synthetic dataset with dense ground truth disparity maps. It contains 35,454 training image pairs and 4370 test image pairs to train the network directly. The end point error (EPE), which is the average disparity error in pixels of the estimated disparity map, was used as an evaluation metric.
2. **KITTI**: KITTI 2015 [25] and KITTI 2012 [29] are real-world datasets with outdoor views captured from a driving car. KITTI 2015 contains 200 training stereo image pairs with sparse ground truth disparity maps and 200 image pairs for testing. KITTI 2012 contains 194 training image pairs with sparse ground truth disparity maps and 195 testing image pairs. The 3-Pixel-Error (3PE), the percentage of pixels with a disparity error larger than three pixels, was used as an evaluation metric.

### 4.2. Implementation Details

We trained the network using the PyTorch [36] framework in an end-to-end manner, with Adam [37] ($\beta_{1}=0.9$, $\beta_{2}=0.999$) as the optimizer. For the Scene Flow dataset, we trained the network with a batch size of 12 using all training sets (35,454 image pairs) from scratch on an NVIDIA GeForce RTX GPU for 120 epochs, and evaluated it using the test set (4370 image pairs). For the preprocessing step, the raw input images were randomly cropped to a size of 256 × 512 and normalized using ImageNet [38] statistics (mean:[0.485, 0.456, 0.406]; std:[0.229, 0.224, 0.225]). The learning rate started at 0.001 and decreased by half at the 20th, 40th, and 60th epochs. We used this dataset for ablation and pretraining for KITTI. For the KITTI dataset, we first fine-tuned the pretrained model for Scene Flow on the mixed KITTI 2012 and 2015 training sets for 1000 epochs. Then, an additional 1000 epochs were fine-tuned on the KITTI 2015 training set. For the mixed KITTI 2012 and KITTI 2015 training sets, input images were randomly cropped to a size of 336 × 960; the initial learning rate was 0.0001 and then decreased by half at the 200th, 400th, and 600th epochs. The same normalization method as the Scene Flow dataset was used for preprocessing. The loss weights for Equation (12) were set to $\lambda_{1}=5$ and $\lambda_{2}=1$, and β value for Equation (13) was set as β=1. The maximum disparity $D_{max}$ of the original input image size was set to 192. The number *M* of MSFF modules in the MSFFNet are set as M=2 which covers the full disparity range of 192 for 1/4 of the input image size, because a single MSFF module covers a disparity range of 24. For the teacher network, GwcNet-g [15] was used, and the cost volume $V_{t}$ was obtained from the teacher network before performing the softargmax regression for the resultant disparity map.

### 4.3. Comparative Result

#### 4.3.1. Scene Flow Dataset

We compared the performance of our proposed method with that of other stereo-matching networks on the Scene Flow test set. Table 2 presents the comparative results of our network and various other 2D/3D convolution-based networks. “Ours” means the EPE of the predicted disparity map of the proposed network. As shown in Table 2, our MSFFNet outperforms all other 2D convolution-based models [10,17,27,28] and some 3D convolution-based models [12,13]. Among the 2D convolution networks, compared with SFFNet [17] which proposed the SFF module, the proposed MSFFNet shows better accuracy with $\left|1.04-1.01\right|/1.04=2.9\%$ lower EPE. Furthermore, among the 3D convolution networks, MSFFNet results in $\left|1.09-1.01\right|/1.09=7.3\%$ better accuracy compared with PSMNet [12] and $\left|1.10-1.01\right|/1.10=8.2\%$ better accuracy compared with StereoNet [13].

**Figure 9:**
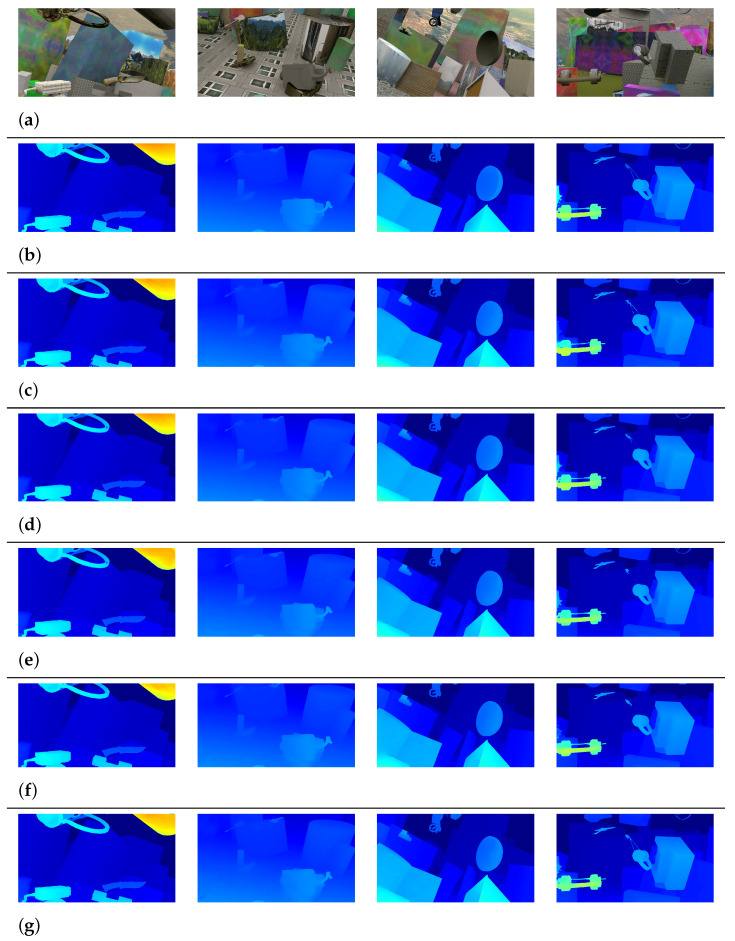
Qualitative comparison on the Scene Flow test set. (**a**) Input Image. (**b**) Ground-truth image. (**c**) PSMNet [12]. (**d**) DeepPruner-Fast [26]. (**e**) SFFNet [17]. (**f**) 2D-MobileStereoNet [28]. (**g**) Ours.

Figure 9 shows a qualitative comparison between the MSFFNet and other networks [12,17,26,28] on the Scene Flow test set. This comparison demonstrates that our network predicts disparity maps comparable to other 2D/3D convolution networks for most regions.

#### 4.3.2. KITTI-2015 Dataset

We compared the performance of our method with other methods on the KITTI 2015 stereo benchmark [25]. Table 3 shows the comparison of various indicators including runtime, 3PE, number of parameters, and FLOPs. In this table, *bg*, *fg* and *all* indicate the percentage of error pixels averaged over the background pixels, foreground pixels, and all ground truth pixels, respectively. In addition, *Noc (%)* and *All (%)* represent the percentages of error pixels for non-occluded regions and all pixels, respectively. As shown in Table 3, our method achieves faster runtime and requires smaller number of parameters and smaller FLOPs with comparable accuracy compared with most 3D convolution-based networks. Specifically, compared with AANet [16] which connects multi-scale networks in parallel, our method has better efficiency with $\left|62-42\right|/62=32.3\%$ faster runtime and $\left|3.9-2.92\right|/3.9=25.1\%$ smaller number of parameters. In addition, compared with AcfNet [18] which directly supervises the predicted cost volume, our method has better efficiency with $\left|480-42\right|/480=91.2\%$ faster runtime and $\left|5.36-2.92\right|/5.36=45.5\%$ smaller number of parameters. Table 3 shows that the proposed MSFFNet is the fastest network using the smallest FLOPs with comparable accuracy among the 2D convolution-based methods. Especially, compared with SFFNet [17], our method has better performance in all indicators with $\left|3.28-2.94\right|/3.28=10.4\%$ better accuracy, $\left|4.61-2.92\right|/4.61=36.7\%$ smaller number of parameters, and $\left|208.21-97.96\right|/208.21=52.9\%$ smaller FLOPs.

**Figure 10:**
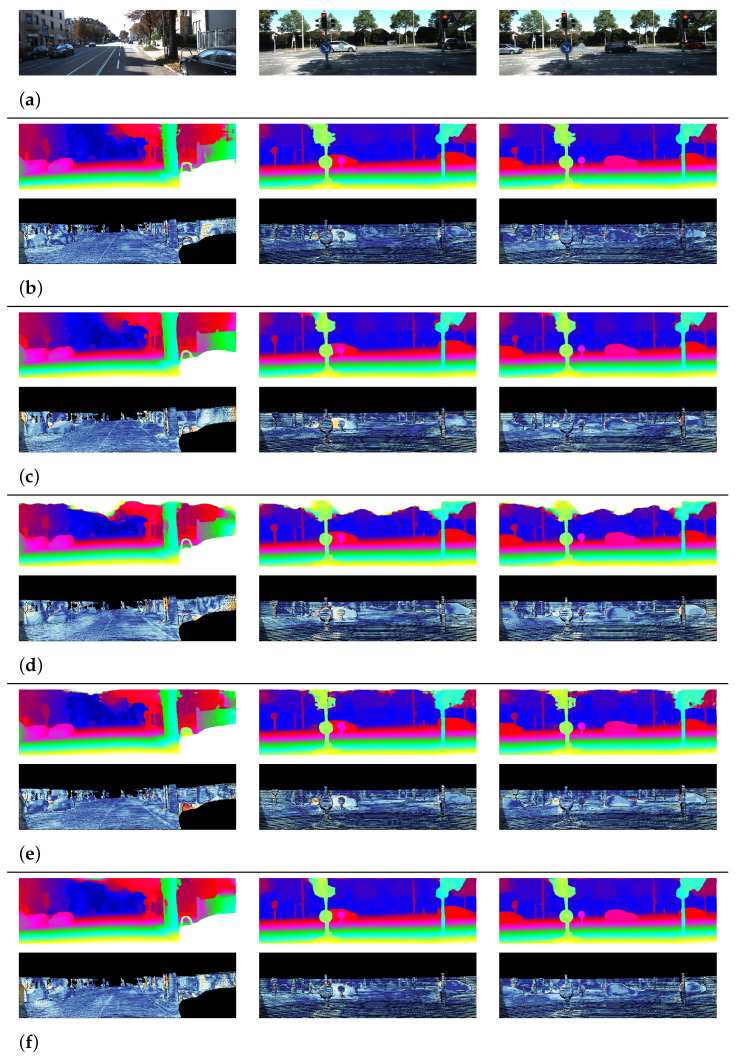
Qualitative comparison on the KITTI-2015 test set. (**a**) Input Image. (**b**) PSMNet [12]. (**c**) DeepPruner-Fast [26]. (**d**) SFFNet [17]. (**e**) 2D-MobileStereoNet [28]. (**f**) Ours.

Figure 10 illustrates some of the qualitative comparison results of the MSFFNet and other networks [12,17,26,28] on the KITTI 2015 benchmark [25]. All figures are reported from the KITTI 2015 evaluation server. The images for each network represent the predicted disparity maps and error maps of the predicted disparity maps for the left input images. In the error maps, a pixel color closer to red indicates a region with a larger error. From this comparison, it is observed that our method generates comparable disparity maps with other methods for various scenes.

### 4.4. Ablation Study

As shown in Table 4, we conducted various experiments with a controlled setup to verify the effectiveness of our proposed method on the Scene Flow dataset. First, we checked the effectiveness of the interlaced concatenation method in our network and then verified the effectiveness of the ACVF loss. All the methods list in Table 4 included the disparity regression loss $L_{d}$ in Equation (6) for training networks as a baseline loss function.

#### 4.4.1. Effect of Interlaced Cost Concatenation

**Figure 11:**
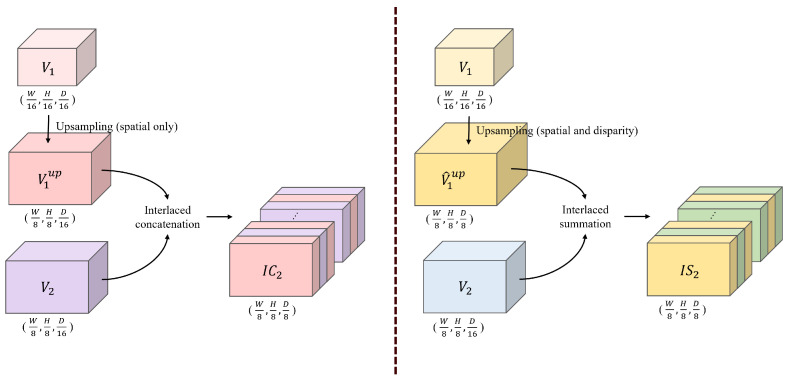
Details of interlaced concatenation and interlaced summation.

To investigate the effectiveness of the proposed interlaced concatenation method defined in Equation (3), we conducted two experiments with the interlaced concatenation method denoted as “Concatenation” and the simple summation method denoted as “Summation” on the Scene Flow dataset, as listed in Table 4. Figure 11 compares the details of these two methods. The proposed concatenation method upsamples the cost volume $V_{i}$ to obtain $V_{i}^{up}$ only in the spatial domain without changing the channel dimension. Subsequently, with the upsampled $V_{i}^{up}$ and $V_{i+1}$, interlaced concatenation is performed using Equation (3) to generate the fused cost volume $IC_{i+1}$. However, the naive summation method upsamples the cost volume $V_{i}$ in both the spatial and disparity domains to produce ${\hat{V}}_{i}^{up}$ which has the same size as the cost volume $V_{i+1}$. Then, $IS_{i+1}$ is obtained by simple summation defined as

(14) $$IS_{i+1}\left(x,y,c\right)=\left\{\begin{array}{c} \begin{array}{cc} {\hat{V}}_{i}^{up}\left(x,y,c\right), & c=2n \end{array}\\ \begin{array}{cc} {\hat{V}}_{i}^{up}\left(x,y,c\right),+V_{i+1}\left(x,y,n\right), & c=2n+1 \end{array} \end{array}\right.,$$

where values for odd disparities in $IS_{i+1}$ are computed by summing the elements of $V_{i+1}$ and those of ${\hat{V}}_{i}^{up}$ for odd disparities. The cost values for even disparities of $IS_{i+1}$ are established using the interpolated cost volume ${\hat{V}}_{i}^{up}$. The “Summation” method uses $IS_{i+1}$ instead of $IC_{i+1}$ for building the cost volumes.

Table 4 shows that the proposed concatenation method achieves a lower EPE (1.098) than the EPE (1.174) of the simple summation method on the Scene Flow test set. The interpolation of $V_{i}$ along the disparity channel smoothens the cost of each odd disparity, which disturbs the cost of $V_{i}$ through a summation. Thus, the proposed concatenation method which maintains the disparity channels of each cost volume generates more accurate results.

#### 4.4.2. Effect of Adaptive Cost-Volume-Filtering Loss

The proposed ACVF loss in Equation (12) consists of two loss components such as $L_{gt}$ using the ground truth disparity map and $L_{kd}$ using the teacher network. To verify the effect of each component, we conducted experiments in which we added each loss $L_{gt}$ and $L_{kd}$ one at a time to the ACVF loss. Using the baseline method where MSFFNet is trained with only disparity regression loss $L_{d}$ in Equation (6), we gradually added only one component of the ACVF loss and then finally added both of them. As shown in Table 4, the addition of each component improves the EPE for the Scene Flow test set compared with the baseline method. Because $L_{gt}$ adds a constraint to the predicted cost volume in terms of reducing bias errors, the EPE of using only $L_{gt}$ is reduced by 0.058. Meanwhile, $L_{kd}$ is helpful for reducing variance errors, EPE is reduced by 0.055. The adaptive addition of both components achieves the best EPE of 1.011 which is reduced by 0.087 compared with the baseline method.

## 5. Conclusions

In this paper, we presented an efficient MSFFNet for stereo matching. The network consists of a series of MSFF modules that efficiently connect multi-scale SFF modules in parallel to generate multi-scale cost volumes. In addition, an interlaced concatenation method is presented to generate a final cost volume which contains the full disparity range by combining the multi-scale cost volumes that include information for only parts of the disparity range. To train the network, an ACVF loss function was proposed to regularize the estimated cost volume using both the ground truth disparity map and the probability distribution map generated from the teacher network with higher accuracy. This loss function adaptively utilizes them based on the error ratio of disparity maps generated from the teacher network and the proposed network. Results of various comparative experiments show that the proposed method is more efficient than previous methods because our network consumes fewer parameters and runs at a faster speed with reasonable accuracy. For example, our network achieves 1.01 EPE with 42 ms runtime, 2.92M parameters and 97.96G FLOPs on the Scene Flow test set. It is 89% faster and 7% more accurate with 45% fewer parameters than PSMNet [12] which is one of the representative 3D convolution-based methods on the Scene Flow test set. Compared with SFFNet [17] which is one of the representative 2D convolution-based methods, our method has better performance with 44% faster runtime, 10% better accuracy, and 36% fewer parameters.

Despite the performance improvements of various aspects, the proposed network has a limitation in that the accuracy of our method is relatively lower than some of heavy 3D convolution-based networks. In future work, the accuracy of our method should be improved by employing a more accurate teacher model to train our network. We believe that our method sheds light on developing a practical stereo-matching network that generates accurate depth information in real time. In addition, our method can be adopted in a variety of applications that require real-time 3D depth information. 

## Figures and Tables

**Table 1:** A comparison of relevant stereo-matching networks.

Network	Cost Volume	Architecture	Cost Volume Filtering	Dataset
MC-CNN [8]	4D	Single scale	X	KITTI
DispNetC [10]	3D	Single scale	X	Scene Flow, KITTI
PSMNet [12]	4D	Single scale	X	Scene Flow, KITTI
AnyNet [14]	4D	Multi scale	X	Scene Flow, KITTI
		Sequential		
AANet [16]	4D	Multi scale	X	Scene Flow, KITTI
		Parallel		
Msmd-net [30]	4D	Multi scale	X	Scene Flow, KITTI
		Parallel		
CompactStereoNet [31]	4D	Multi scale	X	Scene Flow, KITTI
		Sequential		
UASNet [32]	4D	Multi scale	X	Scene Flow, KITTI
		Sequential		
SFFNet [17]	3D	Single scale	X	Scene Flow, KITTI
AcfNet [18]	4D	Single scale	O	Scene Flow, KITTI
CDN [19]	4D	Single scale	O	Scene Flow, KITTI
2D-MobileStereoNet [28]	4D	Single scale	X	Scene Flow, KITTI

**Table 2:** Comparison of EPEs of various methods on the Scene Flow test set.

3D Conv. Models						2D Conv. Models				
PSMNet [12]	StereoNet [13]	Gwc-Net [15]	DeepPruner-Fast [26]	AANet [16]	AcfNet [18]	DispNet-C [10]	CRL [27]	SFFNet [17]	2D-MobileStereoNet [28]	Ours
1.09	1.10	0.79	0.97	0.87	0.92	1.68	1.32	1.04	1.14	1.01

**Table 3:** Quantitative comparison results on the KITTI 2015 test set.

Method	Network	Runtime	Noc (%)			All (%)			Params	FLOPs
			bg	fg	All	bg	fg	All		
3D conv. method	Content-CNN [9]	1000 ms	3.32	7.44	4.00	3.73	8.58	4.54	0.70 M	978.19 G
	MC-CNN [8]	67,000 ms	2.48	7.64	3.33	2.89	8.88	3.89	0.15 M	526.28 G
	GC-Net [11]	900 ms	2.02	3.12	2.45	2.21	6.16	2.87	2.86 M	2510.96 G
	PSMNet [12]	410 ms	1.71	4.31	2.14	1.86	4.62	2.32	5.36 M	761.57 G
	StereoNet [13]	15 ms	-	-	-	4.30	7.45	4.83	0.31 M	-
	GwcNet-g [15]	320 ms	1.61	3.49	1.92	1.74	3.93	2.11	6.43 M	-
	GANet-15 [39]	1500 ms	1.40	3.37	1.73	1.55	3.82	1.93	-	-
	DeepPruner-Best [26]	182 ms	1.71	3.18	1.95	1.87	3.56	2.15	7.39 M	383.49 G
	DeepPruner-Fast [26]	64 ms	2.13	3.43	2.35	2.32	3.91	2.59	7.47 M	153.77 G
	AANet [16]	62 ms	1.80	4.93	2.32	1.99	5.39	2.55	3.9 M	-
	AcfNet [18]	480 ms	1.36	3.49	1.72	1.51	3.80	1.89	5.36 M	-
2D conv. method	DispNetC [10]	60 ms	4.11	3.72	4.05	4.32	4.41	4.34	42.43 M	93.46 G
	CRL [27]	470 ms	2.32	3.68	2.38	2.48	3.59	2.67	78.21 M	185.85 G
	SFFNet [17]	76 ms	2.50	5.44	2.99	2.69	6.23	3.28	4.61 M	208.21 G
	2D-MobileStereoNet [28]	400 ms	2.29	3.81	2.54	2.49	4.53	2.83	2.23 M	125.58 G
	Ours	42 ms	2.35	4.58	2.72	2.53	4. 99	2.94	2.92 M	97.96 G

**Table 4:** Ablation study results of the network architecture and the adaptive cost-volume-filtering loss on the Scene Flow test set.

Network Architecture		Adaptive Cost Volume Filtering		EPE
Concatenation	Summation	$+L_{\mathrm{gt}}$	$+L_{\mathrm{kd}}$	
	✓			1.174
✓				1.098
✓		✓		1.04
✓			✓	1.043
✓		✓	✓	1.011

## Data Availability

Not applicable.

## Abbreviations

The following abbreviations are used in this manuscript:

CNN	Convolutional Neural Network
SFF module	Sequential Feature Fusion module
SFFNet	Sequential Feature Fusion Network
MSFF module	Multi-scale Sequential Feature Fusion module
MSFFNet	Multi-scale Sequential Feature Fusion Network
ACVF	Adaptive Cost Volume Filtering
EPE	End Point Error
3PE	3-Pixel Error
FLOPs	FLoating point Operations Per Second
